# Global Vaccine Hesitancy Segmentation: A Cross-European Approach

**DOI:** 10.3390/vaccines9060617

**Published:** 2021-06-08

**Authors:** Almudena Recio-Román, Manuel Recio-Menéndez, María Victoria Román-González

**Affiliations:** Department of Economy and Business, University of Almería, Carretera de Sacramento s/n, 04120 Almería, Spain; mrecio@ual.es (M.R.-M.); mvroman@ual.es (M.V.R.-G.)

**Keywords:** vaccine hesitancy, segmentation, social marketing

## Abstract

Vaccine-preventable diseases are global mainly in a globalized world that is characterized by a continuous movement of people and goods across countries. Vaccine hesitancy, the reluctance or refusal to vaccinate despite the availability of vaccines, is rising worldwide. What if the problem of vaccine hesitancy could be most effectively managed when treated globally rather than on a national or regional basis? What if a global vaccine-hesitant segment exists and the differences among countries are not so significant? Based on the Global Marketing Strategy paradigm, this paper shows that seven different cross-European segments exist based on the beliefs, attitudes, and behaviors collected in 28 European countries. These pan-European segments are differentiable (people in those segments have similar characteristics that are visibly dissimilar from the ones in other segments) and actionable (organizations would be able to propose interventions to the hesitant segments based on their profiles). With segmentation being the starting point of many public health intervention strategies for avoiding vaccine-hesitancy, the results recommend moderating the full-adaptation strategy that follows the “context matters” principle suggested by several political and public health international organizations. Embracing a more standardized strategy will allow the development of better services and strategies that support and enable desirable vaccination behaviors.

## 1. Introduction

Vaccine hesitancy—the reluctance or refusal to vaccinate despite the availability of vaccines- is rising worldwide. Even though vaccines are considered one of the most important achievements of public health, preventing an estimated 2.5 million deaths each year worldwide and reducing disease-specific treatment costs [1], the World Health Organization (WHO) identified vaccine hesitancy as one of the top ten global health threats of 2019 [2].

Vaccine hesitancy is not a new trend. Since 1795, when Edward Jenner published the book titled “*An Inquiry into the Cowpox*”, vaccination has become a mainstream medical practice all over the world [3]. Nevertheless, from their beginnings, inoculations have had their detractors. The earliest ones were men of the church. They reasoned that infectious diseases were a God-given fact of life and death. Some medical doctors, those that were earning a lot of money from useless but lucrative cures, also enrolled in the anti-vaccination movement early [3]. Vaccination was associated with diverse hazards including tuberculosis, cancer, madness, blood poisoning, and syphilis [4]. From the middle of the 19th century, Great Britain and the countries under its influence made vaccination compulsory. Parents that refused to inoculate their children were sent to prison. The results were disastrous, with social riots that ended with the abolishment of the acts that made vaccination compulsory in 1909. The lessons provided by these first experiences were clear: the risks of vaccination must not be silenced, and compulsory vaccination was not the answer to the lack of public confidence in vaccines.

Nowadays, vaccine doubters are increasing in number. In several Western countries, diseases that had nearly been eradicated are coming into sight again due to their vaccine rates weakening. Vaccine refusal has been increasing in many EU member states [5]. Between 2000 and 2019, the uptake of measles-containing-vaccine first-dose has decreased in 12 EU member states [6]. Moreover, 14 EU member states were below the immunization rate threshold of 95% that was required to achieve herd immunity in 2019 [7]. The same situation could be found in seasonal influenza and other infectious diseases [5]. The last episode of vaccine hesitancy is related to the COVID-19 pandemic. Some people are afraid that the pace of both scientific review and vaccine control could compromise safety. Differences in acceptance rates rage enormously from country to country around the world [8,9]. Governments, public health officials, pharmacy companies, and other stakeholders are worried about people’s willingness to receive vaccines when appropriate [10].

Vaccine-preventable diseases are global in nature. National immunization programs would benefit from coordinated analysis, action, and control to combat cross-border health threats [11]. In order to avoid the negative consequences of the vaccine hesitancy across the general population, it is necessary, first, to determine population sub-groups that adopt that behavior and, second, to reduce any fear or concern and manage the demand for vaccines [12]. Organizations such as governments and public health services face their particular bundle of distinctive challenges. Based on the premise that context matters, the scientific literature has mostly identified a set of key processes that could overcome hesitancy barriers and would enhance vaccine uptake on a nation-by-nation basis [13]. What if the problem of vaccine hesitancy could be most effectively managed when treated globally rather than on a national or regional basis? What if a global vaccine-hesitant segment exists and the differences among countries are not so significant?

Independent of the pro-vaccination strategy to follow, audience targeting and segmentation strategy are the keys to success [13]. People’s attitudes, values, and observed behaviors are the basis to obtain insights for better targeting the intervention mix to maximize vaccine uptake [14]. Organizations that work in worldwide markets face the dilemma of whether to segment markets on a country-by-country basis or to treat the different segments that exist with adapted value propositions, or whether to target one or more similar segments in a standardized way with the same intervention mix—also known as cross-market segmentation—[15]. Public health has used health education, health promotion, and social marketing as effective tools for influencing behavior in the fight against several communicable and non-communicable diseases [16]. Identifying global vaccination segments, to the extent that they exist, would allow designing and implementing a more efficient and effective public health intervention strategy, since cross-country segments would be targetable with similar activities.

To the best of our knowledge, the Global Marketing Strategy (GMS) paradigm has not been applied to fight against infectious diseases by supranational political institutions and health organizations. GMS proposes that a global organization must standardize its marketing programs across countries as much as possible, mainly concerning its product offering, promotional mix, price, and channel structure [17]. Most of the guidelines that international and national health organizations and governments suggest following when designing a social marketing vaccination strategy are not global [16,18,19,20,21,22]. Based on the principle of “context matters”, they suggest that different countries must design and implement marketing plans adapted to their “unique” characteristics. It means they have adopted the traditional form of international segmentation known as a multidomestic strategy. Several researchers argue that global marketing strategy plays a critical role in determining an organization’s performance in the global market [17,23,24,25]. Hence, these international political institutions and health organizations that do not apply—or recommend not to apply—the GMS paradigm could be achieving suboptimal outcomes on developing better services and strategies that support and enable desirable vaccination behaviors.

The standardized strategy accompanying the GMS approach enhances performance in sectors in which competition is global in scope [17]. These major benefits are mainly obtained through economies of scale and scope, consistency in dealing with the target groups, and the ability to exploit good ideas at a global scale. The global pharmaceutical industry produces vaccines. The pharmaceutical industry is comprised of some major multinational companies operating in a highly global competitive market that has experienced significant growth during the past two decades. Pharmaceutical revenues worldwide totaled USD 1.25 trillion in 2019 [26]. Vaccine hesitancy is also fueled by misinformative campaigns promoted by political, religious, and social organizations on a global scale [27]. Misinformation leads to mistrust in public health organizations and encourages antiscience sentiments [28]. This fake information is transmitted worldwide at lightning speed in a single click. Responding to the global threat posed by vaccine hesitancy with local intervention actions seems not to be enough.

This study is designed to answer two research questions. The first is to confirm whether there are homogeneous segments based on vaccination attitudes, beliefs, and behaviors across European countries. Cross-national segmentation is challenging when cultural and economic differences influence customer preferences [29]. Our study, which analyzes cross-national market segments of individuals with respect to the acceptance of vaccination in 28 European member countries, provides valuable insights into international political and health organizations, companies, practitioners, and academics. The second question is whether the pan-European segments that exhibit higher hesitancy are differentiable (the people in those segments should have similar needs that are visibly dissimilar to the needs of the people in other segments) and actionable (organizations have to be able to propose interventions to the hesitant segments).

## 2. Materials and Methods

The data comes from the EUROBAROMETER survey 91.2 that was carried out between the 15th and the 29th of March 2019, at the request of the European Commission [30]. The dataset was accessed through GESIS (Leibniz-Institute für Sozialwissenschaften, University of Cologne, Germany). The EUROBAROMETER is part of wave 91.2 and covers the population of the respective nationalities of the European Union member states, residents in each of the member states, and aged 15 years and over. In these countries, the survey covers the national population of citizens of the respective nationalities and the population of citizens of all the European Union member states that are resident in those countries and have a sufficient command of one of the respective national language(s) to answer the questionnaire. The basic sample design applied in all states is a multi-stage random one.

The following table (Table 1) shows the sample size in each country and the total population aged 15 or more years.

For answering whether cross-European vaccination segments exist based on vaccination attitudes, beliefs and behaviors, we applied a factor-cluster segmentation approach. We selected all the variables shown in Table A1. From the 46 variables, 44 were coded as binary, and 2 as polytomous (the one that asked if “*vaccines are effective*” and the one that asked “*the most trusted info source*”). For performing the correct association matrix for the factor analysis—using tetrachoric or polychoric correlations, when appropriate—we used the “polycor” package from R [32]. Aiming to reduce the complexity of the observed data to a more limited set of components and to avoid multicollinearity problems, we computed a principal component analysis (PCA) using the “psych” package from R. Zero frequency cells were replaced by 0.5 considering Yate’s correction for continuity [33]. Using the scores for the resulting components, we clustered them choosing the best clustering method between hierarchical methods, K-Means, and PAM considering three internal measures of clustering validation: Connectivity, Dunn, and Silhouette. Attending to the compactness, separation, connectivity, and interpretability of the solution, we chose the optimal number of segments, described and labeled them. For testing whether the pan-European segments that exhibit higher hesitancy were differentiable, we calculated segments’ means differences applying an ANOVA (Tukey HSD). To check if the found segments were cross-European, we ran a Bayesian multilevel multinomial analysis. Once we confirmed that there were no differences between countries for the clustering solution, we performed a multinomial logit regression for testing the actionability of the segments. For better interpreting these results, we used marginal effects.

## 3. Results

PCA analysis results are shown in Table A2 (Appendix B). To determine the number of components we ran a parallel analysis [34] using the “psych” package from R. The results of the parallel analysis suggested that 14 components explaining 66% of the total variance might be most appropriate—RMSR = 0.05 and fit based upon off-diagonal values = 0.94–0.0.

For interpreting the components, we used the Varimax rotated component analysis matrix depicted in Table A2 (Appendix B). Our cutoff point for interpretation purposes was all loadings ±0.4 or above [35,36,37]. Considering the loadings, we named the components as depicted in Table 2.

In sum, we obtained 14 components out of the 44 original variables that explained 66% of the total variance. The two most important ones, in terms of the total variance explained individually, were related to the belief that infectious diseases kill (C2, 8% of the total variance) and vaccines are important to fight them (C1, 9% of the total variance). On the other hand, if we consider all the components that were connected with the information (C4, C6, C8, C9, C10, and C13) summed up the highest proportion of the total variance explained (23%). The rest of the components could also be gathered into three different groups: the first connected with the vaccination status (C5 and C11, 9% of the total variance); the second related to the knowledge about vaccines (C3) and vaccination (C12), which together explained 9% of the total variance; and, finally, two components (C7 and C14) linked with the international level at which the vaccination programs should be managed (7% of the total variance explained).

**Figure 1:**
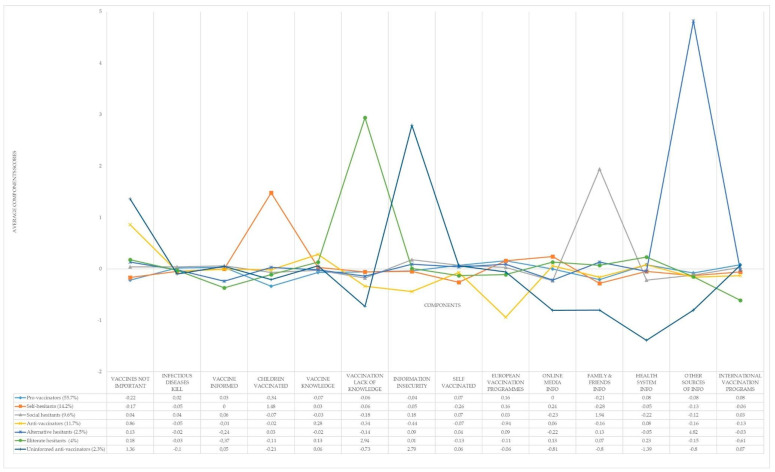
European sentiments towards vaccines. Behavioral segments profile.

Using the factor scores for each of the fourteen components obtained in the previous step for all the interviewees, we proceeded to analyze the different behavioral segments that existed towards vaccination in the European Union. For choosing the best clustering method we used the package “clValid” from R [39]. Hierarchical methods performed better than K-Means and PAM for the three internal measures of clustering validation used (Connectivity, Dunn, and Silhouette). Considering the compactness, separation, connectivity, and interpretability, the seven-cluster solution performed the best. Figure 1 depicts the results of the hierarchical clustering approach (using the squared Euclidean distance and the Ward method). The characteristics of each of the found segments were:

1. **Pro-Vaccinators** (55.7% of the sample). It was the most numerous European segment. Following the segment profile represented in Figure 1, people for whom vaccines were the most important for avoiding the negative effects of infectious diseases formed it (in Table A3, Appendix C, we saw that mean differences with all the other segments were statistically significant). It belonged to the group of segments that answered that they felt better informed about vaccines, but the information received was highly insecure. Attending to how the “Information insecurity” component was composed, we saw that it had three significant loadings—the answers “None (SPONTANEOUS)” and “DK” to the question “If you were looking for information about vaccination, which of the following sources would you consult?”, and the high importance of the option “Family” when responding the question “And which of the following sources do you trust the most for information on vaccination?”—It portrays a component with the family as the most important source of information about vaccination, under the feeling of insecurity about any information source related to this issue. All the fake news that is present in the information environment is affecting the perception of knowledge, even in the pro-vaccinators segment. Insecurity about information affects the perception of knowledge. Therefore, the Europeans that belonged to this segment had the lowest scores on knowledge about vaccines and vaccination. They were vaccinated in the last five years. Their most preferred source of information was the Health System Info. They agreed that either European or international organizations should manage vaccination programs globally.
2. **Self-hesitants** (14.2% of the sample). It was the second segment that made a point on jabs to avoid infectious diseases. They shared a profile with Pro-vaccinators in relation to the information: they had information but not knowledge about vaccines, and were not personally vaccinated due to information insecurity. They had no doubts about their child’s vaccination (see in Figure 1 that they had the highest score of all segments) but they did when they were inoculated. That was why they were labeled as “Self-hesitants”. Their favored source of information was Online Media Info, followed by the Health-System Info.
  They agreed that vaccination programs should be managed by international organizations, with the European authorities being the most preferred ones.
3. **Social-hesitants** (9.6% of the total sample). This segment displayed a medium-range position on the importance of vaccines in avoiding infectious diseases. The respondents replied having been inoculated in the past five years at the same level as the Pro-vaccinators but their hesitance affected their child’s vaccination. They declared themselves to be informed but with a feeling of lack of knowledge about vaccines and vaccination. This led them to information insecurity. They did not trust Online Media Info nor Health System Info preferring, by far, their relatives as the main information source. That is why we called them Social-hesitants. They slightly preferred that international organizations managed the vaccination programs instead of the European authorities.
4. **Anti-vaccinators** (11.7% of the sample). They showed the lowest confidence in vaccines of all the segments. They declared themselves to be well informed and with top knowledge on vaccines but not on vaccination. They did not feel insecure about the information received. Their unconfident belief caused them not to be open about taking vaccines, but they did not show the same behavior for their children. Their preferred sources of information were Online Media Info and Health System Info. A remarkable characteristic of this segment was that they did not trust international organizations for managing the vaccination programs, mainly favoring the European ones.
5. **Alternative-hesitants** (2.5% of the sample). The hesitancy for this group was mainly based on the lack of confidence in Online Media Info and Health System Info. Otherwise, they felt comfortable with Family and Friends Info and showed an absolute preference for Other Sources of Info. In consequence, the reported information insecurity was also high. They portrayed a lack of knowledge about vaccines and vaccination. They shared with the other hesitant groups the lack of confidence in vaccines but, surprisingly, they and their children were among the top segments that had taken vaccines in the last five years.
6. **Illiterate-hesitants** (4% of the sample). This group share with other hesitants their lack of confidence in vaccines. Their most noteworthy characteristic is that they declared themselves not to be well informed about vaccines. Their vaccination status in the past five years was in the medium range of all groups. The most liked source of information was the Health System Info, closely followed by Online Media Info and Family and Friends. They showed a complete lack of confidence in international organizations when managing the vaccines’ programs. European authorities were also not well considered.
7. Uninformed Anti-Vaccinators (2.3% of the sample). The first distinctive attribute is that they had the lowest score in vaccine trust. Accordingly, they showed the lowest score in believing that infectious diseases kill. They declared themselves to be vaccine informed and to have an average knowledge on vaccines. Nonetheless, they had an absolute lack of knowledge about vaccination. The insecurity felt by this group about the information received was also the highest of all the segments found. It was so high that they did not trust any source of information. They rather preferred that the international organization would manage the vaccination programs. All these sentiments produced low vaccination rates among their children.

For answering the question of whether countries affected the results obtained we ran a Bayesian multilevel multinomial analysis using STAN [40]. The dependent variable was the one with the resulting segments (reference category: Pro-Vaccinators) and as independent variables, we considered each of the fourteen principal components obtained. In doing so, we checked to what extent did the log-odds varied between countries computing an unconditional mean model and calculating the intraclass correlation coefficient (ICC). The parameters used for running the Hamiltonian Monte Carlo sampler algorithm (MCMC) were 2500 warmup iterations, 4 chains, 10,000 iterations per chain, and initials values taken at random. The solution converged (Rhat = 1.0) and the results are shown in Table 3.

The ICC quantifies the degree of homogeneity of the outcome within countries. The ICC represents the proportion of the between-countries variation $var\left(u_{0j}\right)$ in the total variation (Equation (1)).

(1) $$ICC=\frac{var\;\left(u_{0j}\right)}{var\left(u_{oj}\right)+\left(\pi^{2}/3\right)}$$

In which, $var\left(u_{0j}\right)$ is the level-2 variance component and $\left(\pi^{2}/3\right)$ refers to the standard logistic distribution, that is, the level-1 variance component. The ICC may range from 0 to 1. ICC = 0 indicates perfect independence of residuals: The chance to pertain to a behavioral segment does not depend on country membership. However, ICC = 1 indicates perfect interdependence of residuals: The segment’s membership only varies between countries. Calculating, we obtained ICC= 0.095. In other words, it means that between countries the differences in the segmentation achieved are negligible. Due to this reason, we performed a multinomial logit model for calculating the relationship between the segments and the principal components found (see Table 4).

From Table 4, we noticed that most of the independent variables—the principal components—are statistically significant in explaining the European segments. To improve the interpretability of the regression coefficients, we used marginal effects. The marginal effect is a measure of the instantaneous effect that a change in a particular explanatory variable has on the predicted probability of the dependent variable when the other covariates are kept fixed [41]. The dependent variable is modeled as follows:

(2) y=E(y/x)+ε,

where E(y/x) is the conditional mean function, x is the vector of explanatory variables and ε is the error term. The conditional mean function is given by:

(3) $$E\left(y/x\right)=F\left(\beta^{\prime }x\right),$$

where F denotes a cumulative distribution function and β denotes the parameters. Therefore,

(4) $$\mathrm{Pr}\left(y=1\right)=F\left(\beta^{\prime }x\right).$$

Marginal effects are obtained by computing the derivative of the conditional mean function given by:

(5) $$\frac{\partial E(y/x)}{\partial x}=\left[\frac{\partial F\left(\beta^{\prime }x\right)}{\partial \beta^{\prime }x}\right]\beta =f\left(\beta^{\prime }x\right)\beta ,$$

where f(.) is the density function that corresponds to the cumulative function F(.). The marginal effects are nonlinear functions of the parameter estimates and levels of the explanatory variables. Hence, they generally cannot be inferred directly from parameter estimates. In this case, we used the R library called “margins”. The results are available in Appendix D, Figure A1, Figure A2, Figure A3, Figure A4, Figure A5, Figure A6, Figure A7, Figure A8, Figure A9, Figure A10, Figure A11, Figure A12, Figure A13 and Figure A14.

**Figure A1:**
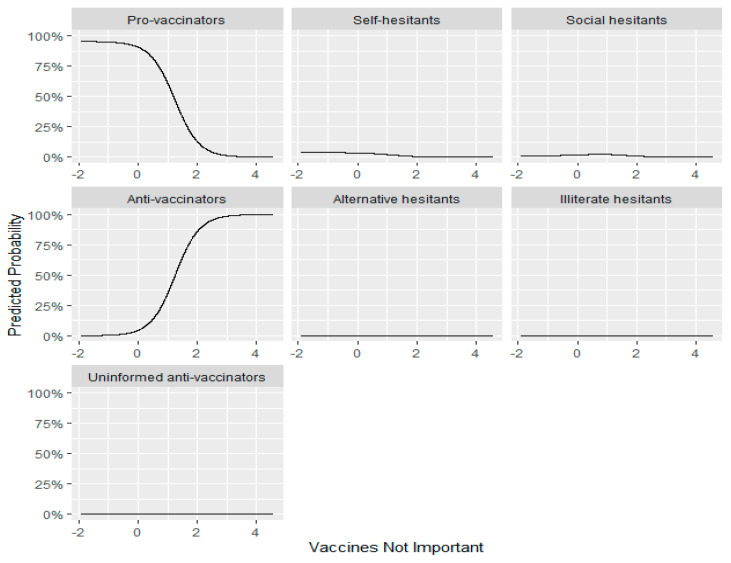
Marginal Effects Multinomial Logistic Regression. Vaccines Not Important.

**Figure A2:**
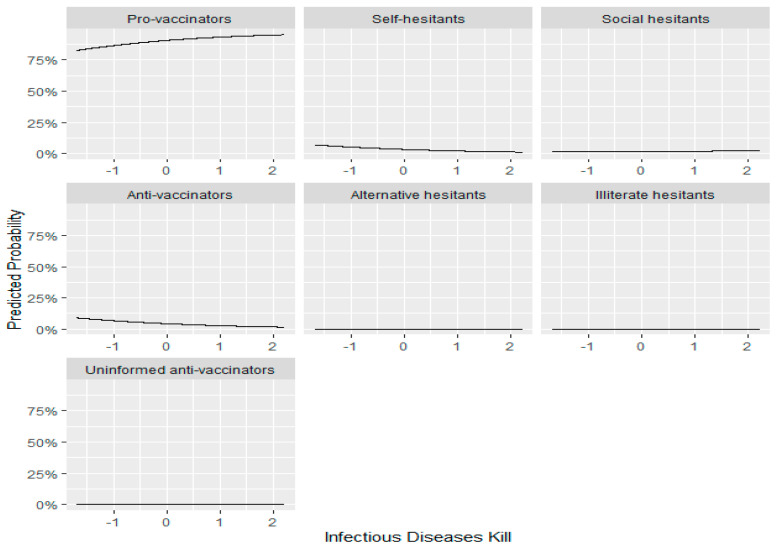
Marginal Effects Multinomial Logistic Regression. Infectious Diseases Kill.

**Figure A3:**
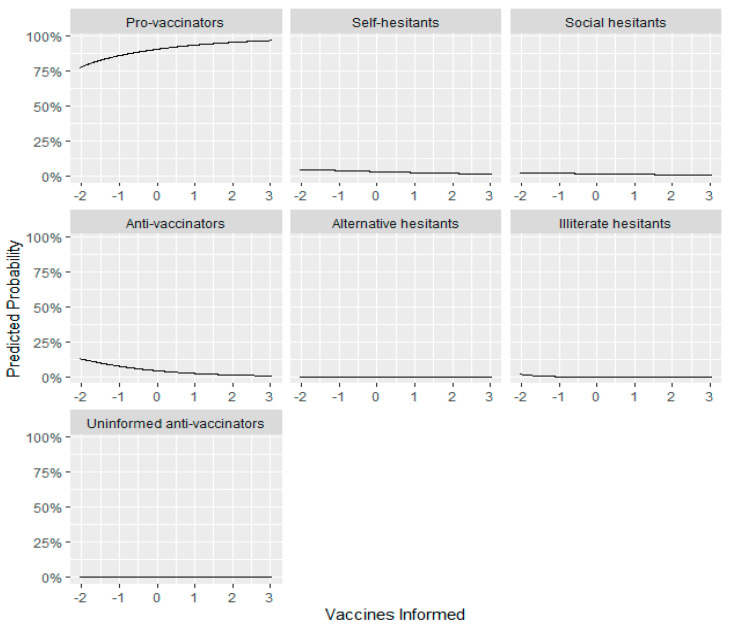
Marginal Effects Multinomial Logistic Regression. Vaccines Informed.

**Figure A4:**
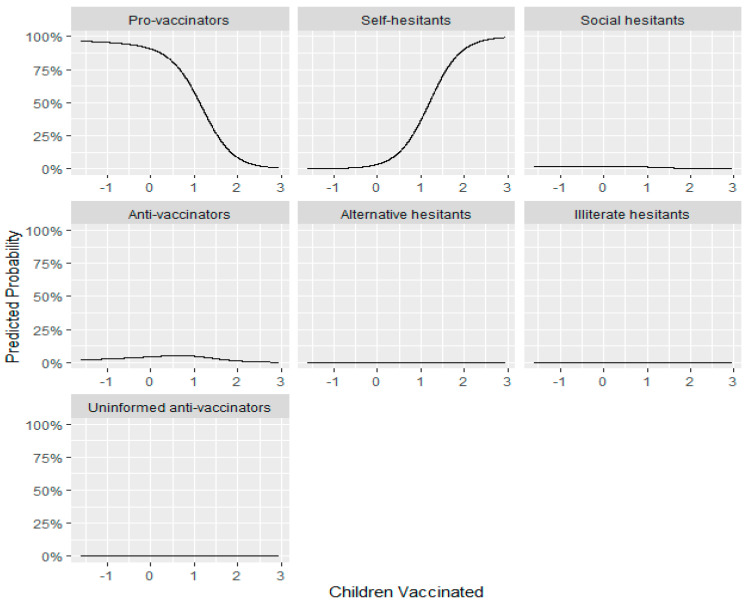
Marginal Effects Multinomial Logistic Regression. Children Vaccinated.

**Figure A5:**
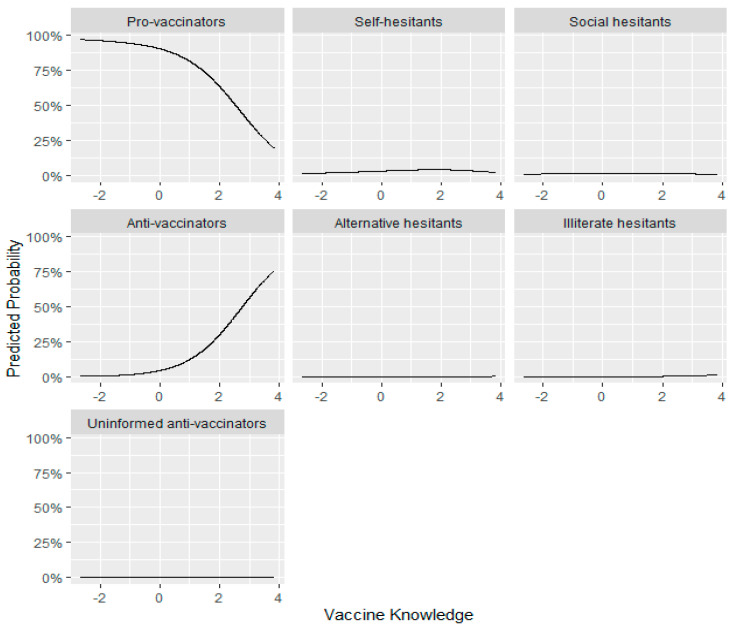
Marginal Effects Multinomial Logistic Regression. Vaccine Knowledge.

**Figure A6:**
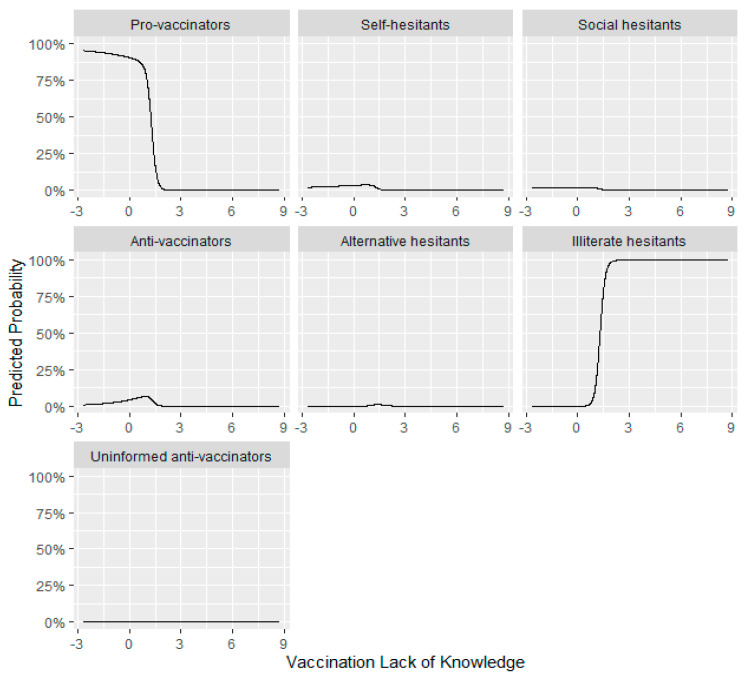
Marginal Effects Multinomial Logistic Regression. Vaccination Lack of Knowledge.

**Figure A7:**
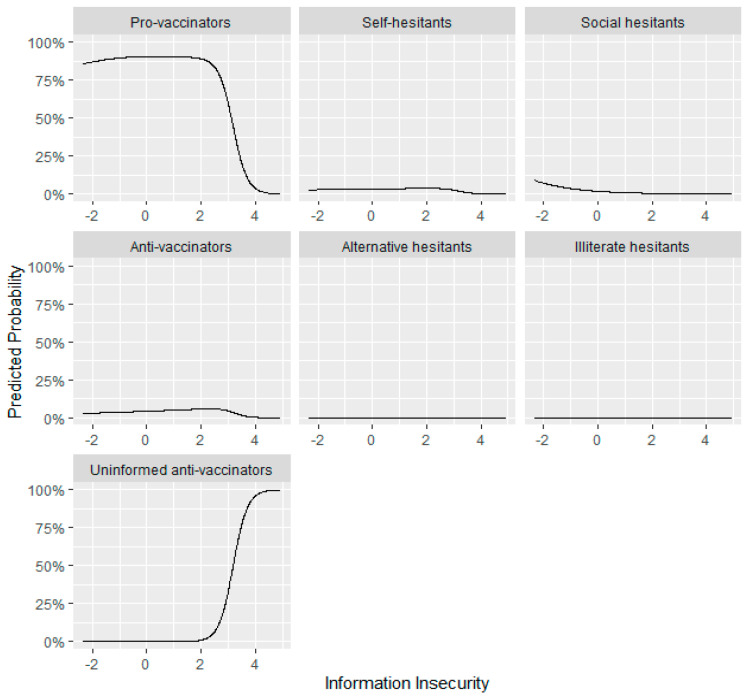
Marginal Effects Multinomial Logistic Regression. Information Insecurity.

**Figure A8:**
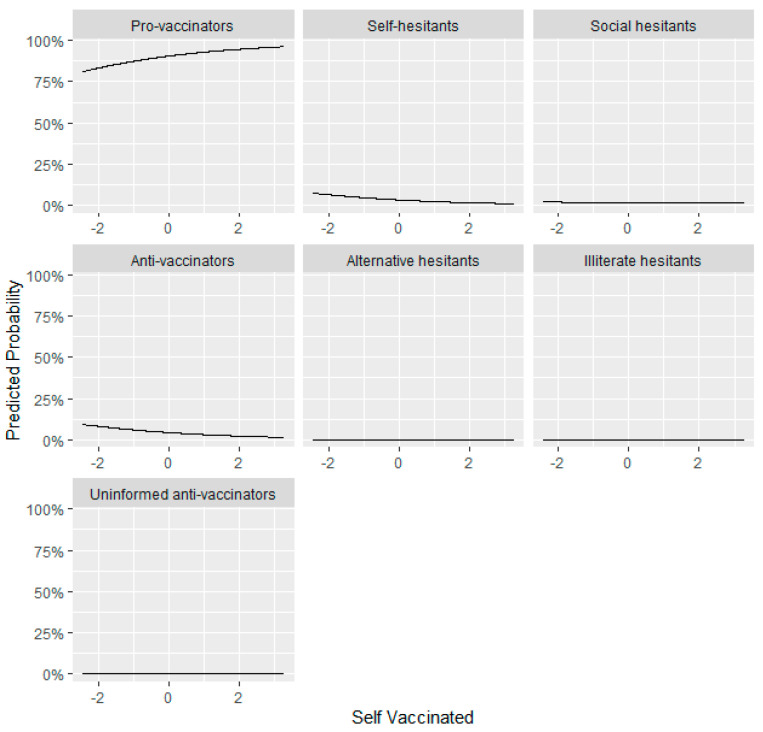
Marginal Effects Multinomial Logistic Regression. Self Vaccinated.

**Figure A9:**
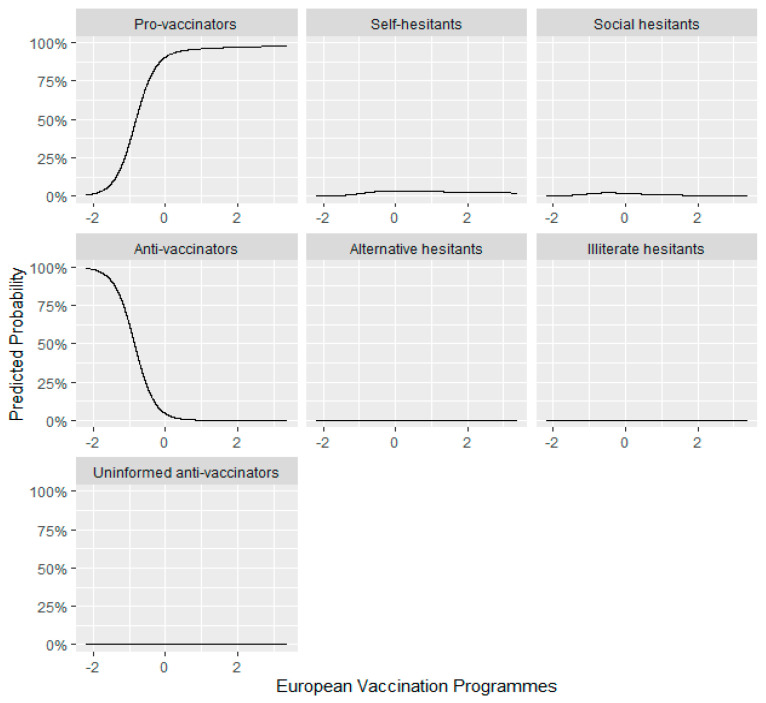
Marginal Effects Multinomial Logistic Regression. European Vaccination Programs.

**Figure A10:**
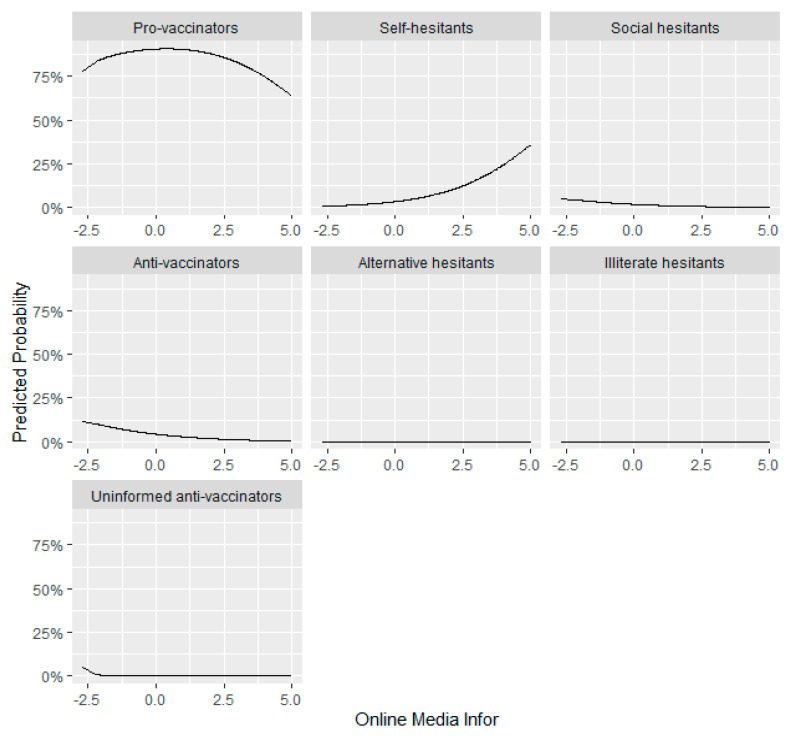
Marginal Effects Multinomial Logistic Regression. Online Media Info.

**Figure A11:**
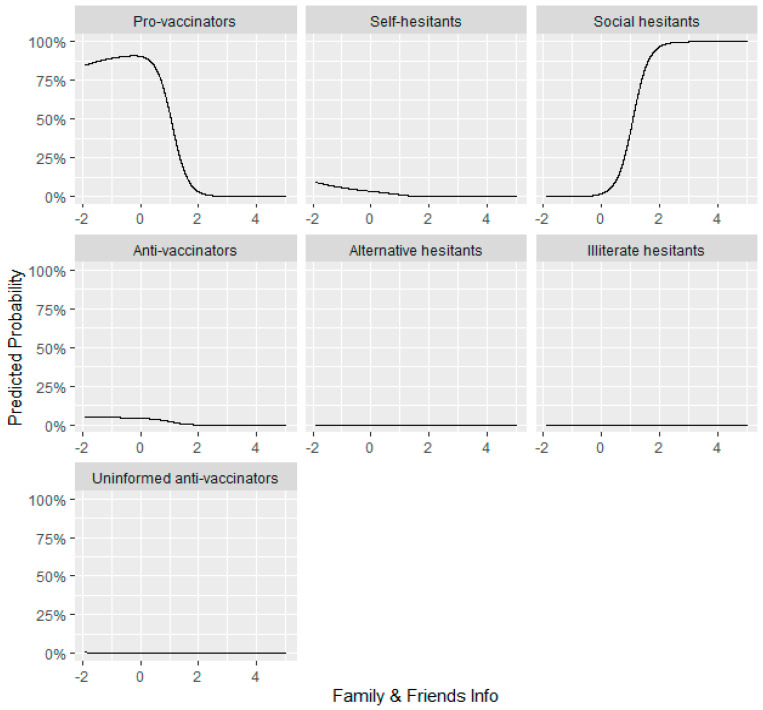
Marginal Effects Multinomial Logistic Regression. Family and Friends Info.

**Figure A12:**
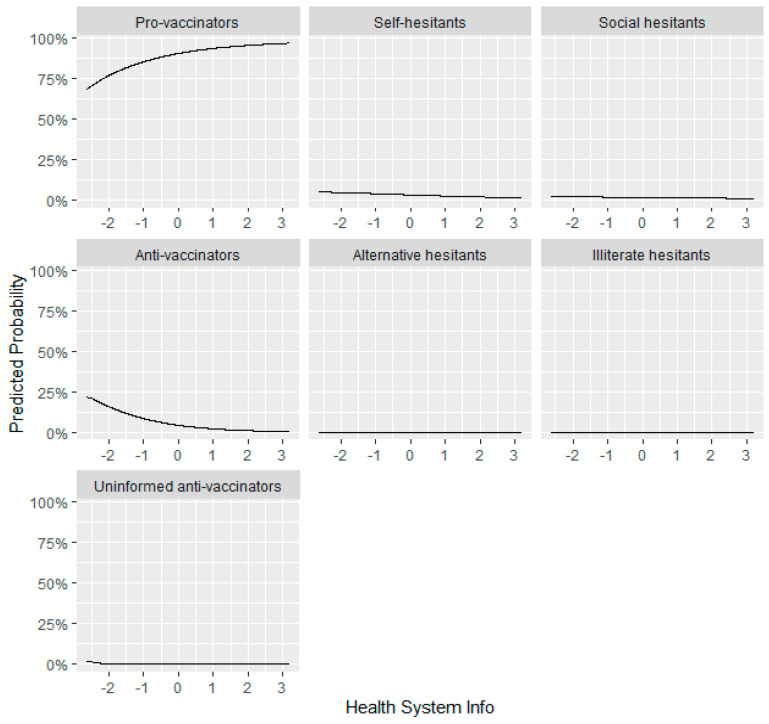
Marginal Effects Multinomial Logistic Regression. Health System Info.

**Figure A13:**
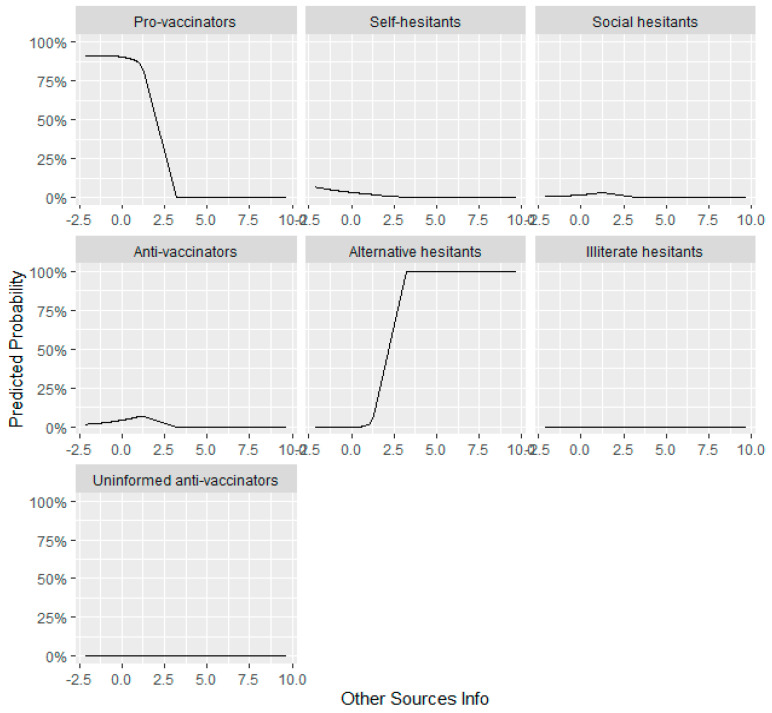
Marginal Effects Multinomial Logistic Regression. Other Sources Info.

**Figure A14:**
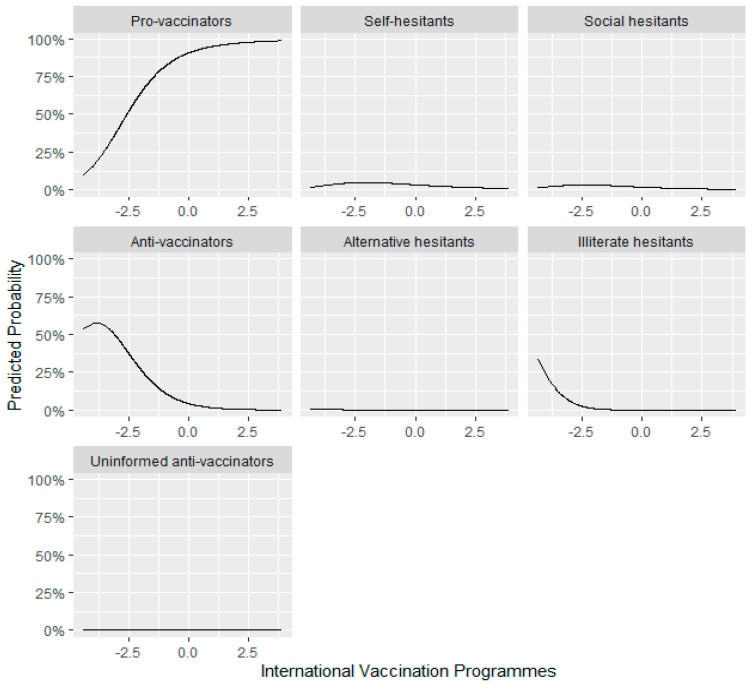
Marginal Effects Multinomial Logistic Regression. International Vaccination Programs.

By simultaneously interpreting the data contained in Table 4 and Figure A1, Figure A2, Figure A3, Figure A4, Figure A5, Figure A6, Figure A7, Figure A8, Figure A9, Figure A10, Figure A11, Figure A12, Figure A13 and Figure A14, we obtained the following results. Pro-Vaccinators and Anti-Vaccinators had differentiated profiles. Furthermore, the predicted probability for belonging to one of these groups showed an inverted shape in the case of “Vaccines Not Important” (Figure A1), “Infectious Diseases Kill” (Figure A2), “Vaccines Informed” (Figure A3), “Vaccine Knowledge” (Figure A5), “‘Self Vaccinated” (Figure A8), “European Vaccination Programs” (Figure A9), “Health System Info” (Figure A12), and “International Vaccination Programs” (Figure A14). Self-Hesitants shared with Anti-Vaccinators the aforementioned reversed profile with Pro-Vaccinators in the case of “Infectious Diseases Kill” (Figure A2), “Vaccines Informed” (Figure A3), “Self Vaccinated” (Figure A8) and “Health System Info” (Figure A12). Moreover, this inverted predicted probability profile between Self-Hesitants and Pro-Vaccinators was also present in the variables “Children Vaccinated” (Figure A4) and “Online Media Info” (Figure A10). Illiterate-Hesitants depicted their main differential characteristic with Pro-Vaccinators in the variable “Vaccination Lack of Knowledge” (Figure A6). The main portrayed difference between Uninformed Anti-Vaccinators and Pro-Vaccinators arose in the component “Information Insecurity” (Figure A7). Social-Hesitants and Pro-Vaccinators differed markedly in “Family and Friends Info” (Figure A11). Finally, the main differential characteristic between Alternative-Hesitants and Pro-Vaccinators rose in ‘Other Sources Info’ (Figure A13). These results corroborated the findings commented when previously describing the behavioral segments’ profiles in Figure 1.

## 4. Discussion

In the previous section, we have presented results that allow answering the two main research questions proposed. First, based on vaccination attitudes, beliefs and behaviors there exist seven different homogeneous segments across the European countries. These pan-European segments are differentiable and actionable.

**Figure 2:**
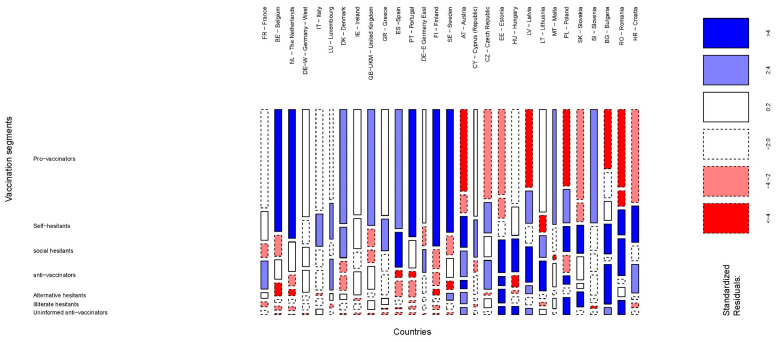
European Attitudes Towards Vaccination. Segments by Country.

In a recent report based on a descriptive analysis of the same survey that we used in this paper, the European Commission stated that “…While in general Europeans have a reasonably high level of awareness and a generally positive attitude towards vaccination, there is considerable variation in knowledge and behavior across countries and between socio-demographic groups” [42] (p. 59). The results in the previous section of this paper depicted that cross-European segments based on attitudes towards vaccination existed. When European countries were considered in order to see if there were any significant variation between them in the segments obtained, we concluded that it was not the case. The geographical differences found by the European Commission could be rather linked to the different proportions in which the cross-European segments were represented in each of the member countries than the non-existence of these homogeneous groups. It can be graphically appreciated in Figure 2. It shows a MOSAIC chart [43] that can be interpreted, in a two-way table, as a grouped bar chart where the width of each bar corresponds to the relative frequencies of the first variable (number of interviews per country) and the height of each bar shows the relative frequencies of the second variable (European segments towards vaccination). Standardized residuals are represented in the chart by shadowing the tiles: those that exceed values 2 and 4 in absolute terms are deep-colored. When it occurs, it means that the found pattern departures from the Equiprobability model (independence between the variables). Statistically speaking, it means that, as the standardized residuals are approximately unit-normal N(0,1), the shadowed areas are those whose individual residuals are significant at 0.05 level (when the value exceeds 2) and 0.0001 level (when the value exceeds 4) [44]. For the shake of clarity, we observe in Figure 2 that Pro-Vaccinators had a statistically significant higher presence in Belgium, the Netherlands, the United Kingdom, Spain, Portugal, Finland, Sweden, Malta, and Slovenia. On the contrary, Pro-Vaccinators showed a statistically significant lower presence in Austria, Czech Republic, Estonia, Latvia, Poland, Slovenia, Bulgaria, Romania, and Croatia. On the other side of the attitudes’ spectrum, Anti-Vaccinators had a statistically significantly higher presence in France, Luxembourg, Austria, Czech Republic, Estonia, Latvia, Lithuania, Bulgaria, Romania, and Croatia. In contrast, Belgium, the Netherlands, Denmark, Spain, Portugal, Finland, Sweden, Hungary, and Poland had a statistically significantly lower presence of Anti-Vaccinators. The rest of the segments and countries could be distinctly exhibited in Figure 2. The differences observed by the European Commission between European countries in their descriptive analysis are due to the different share that the seven cross- European segments had in each of the territories. Nevertheless, the profile of any of the individuals that belong to a segment remains homogeneous to the rest of the individuals that also pertain to the same segment, independently of the European country under study.

The seven segments obtained overcome the traditional pro-vaccine versus anti-vaccine approach. Between these two extreme poles, five other vaccine-hesitant behaviors were found. As we have seen, the individuals that comprised these segments can retard, be averse but still uptake, or decline some or all vaccines. Furthermore, the process followed to obtain the segments avoided the negative connotations associated with the terms “anti-vaccine” and “vaccine-hesitant”. When conducting research about vaccine hesitancy we have to take into account that even those individuals that present the most radical profile do not recognize themselves as “anti-vaccine” [45]. Thus, in the survey that we used, the individuals were first asked about beliefs, attitudes, and behaviors about vaccines and vaccination, and then, after applying sound statistical techniques, we found the segments that were labeled taking into account their different profiles.

From the private companies’ management perspective, the findings presented are important. Infectious diseases are global by their nature, mostly in a global economy characterized by a continuous flow of goods and persons between countries. The pharmacy industry and vaccines are also global [46]. Therefore, only if different client behaviors exist that are profitable for the private companies to fulfill with adapted marketing strategies, it would be justified not to follow a Global Marketing Strategy (GMS) approach. Nowadays, for instance, there is more convergence in demand for newer vaccine types and more divergence in demand for mature and combination vaccine types. For the latter ones, adaptations to existing vaccine presentations and packaging are required and increasingly requested. Manufacturers benefit from these distinct presentations, as they prevent parallel trade between high-income countries and low-income countries enable manufacturers to pursue multiple pricing strategies. The existence of cross-European vaccination segments offers additional evidence for private companies when deciding on the continuum that goes from the full standardization to the full adaptation of marketing strategy.

For social marketers (v.gr. European Commission, International Health Organizations, governments, and health authorities), behavioral segmentation is key for success when choosing the target audience and developing different marketing strategies for selected population segments. Social marketing has been long employed in designing, implementing, and evaluating public health programs in the fight against several forms of communicable diseases [47,48,49]. Moreover, the GMS approach remains valid: adaptation is mainly recommended when there are behavioral differences between the segments that when taken into action produce better results. Our Bayesian multilevel multinomial analysis showed that there were no statistically significant differences for the clusters when considering the 28 countries that formed the European Union. Hence, public organizations that apply a standardized marketing strategy across countries will obtain better outcomes [17,23,24,25]. The Anti-Vax industry is applying these standardized marketing strategies in their disinformation campaigns obtaining better results than the public institutions that are fighting against them with adapted marketing actions [50].

Information is key in both the understanding of the vaccine up-taking decision process and the characterization of the different segments around vaccination. From the results obtained, we noticed that the components related to the different sources of information summed up the highest proportion of the total variance explained (23%). From Table A1 we knew that the most trusted source of information was a general practitioner, a doctor, or a pediatrician (79.1% of the total responses). Several investigations showed that a significant share of health care providers is vaccine-hesitant [51,52,53,54,55]. Even though few health care providers are openly against vaccines, many of them find conversations about vaccines with vaccine-hesitant people to be difficult and unproductive [52]. This has to be a matter of concern for public health authorities. The results of our research also showed that some segments declare a lack of knowledge around vaccines and/or vaccination (Uninformed Anti-Vaccinators and Social-Hesitants). Fighting against the lack of knowledge has to be a priority as a starting point. Nevertheless, lessening the growth of vaccine hesitancy requires not only to communicate information about vaccine efficacy and safety but engaging with the problems expressed by the citizens in an empathically two-way communication strategy [56,57]. Furthermore, it also means sending the tailored messages through the right communication channels. As we saw in the Results section, Pro-Vaccinators and Illiterate-Hesitants preferred the Health system info, Self-Hesitants and Anti-Vaccinators favored social media Info, Social-Hesitants chose their relatives as the main information source, Alternative-Hesitants indicated other sources of information and, finally, Uninformed Anti-Vaccinators felt such high insecurity about the information received that they did not place trust in any source of information.

Trust in international organizations positively influences people’s willingness to adopt recommended behavior [58,59]. As we saw, the level of trust in the international organizations to carry on the vaccination programs varied among segments. Pro-vaccinators trusted in European as well as international organizations for managing vaccination programs. Self-Hesitants and Alternative-Hesitants preferred the European ones. Reversely, Social-Hesitants and Uninformed Anti-Vaccinators favored International Organizations. Anti-Vaccinators and Illiterate-Hesitants did not trust any international organization in the management of the vaccination programs, mainly the European ones. Therefore, depending on the target audience, the source of the tailored communication campaign has to be adequately selected to be trusted.

Once that we know that global vaccine hesitancy segments exist across, Europe some other challenges arise. For instance, in Europe immunization programs are a national competence with vaccination schedules that vary across the different territories. We face the GMS dilemma of global consumers with local organizations. The European Commission and the Member States need to put in place coordinated operational guidelines for overcoming infrastructural and legal barriers through more standardized vaccination management. In this regard, two European initiatives taken in 2018 [11,60] accomplished an actions’ framework that was undertaken by the Commission, with the collaboration of the Member States, under three key pillars: (1) tackling vaccine hesitancy and improving vaccination coverage; (2) sustainable vaccination policies in the EU; (3) EU coordination of and contribution to global health. The roadmap for the implementation of actions contained in these two European initiatives fixes several challenges that a global social marketing strategy for reducing vaccine hesitancy must face because immunization programs are a national competence.

Finally, more study is required to understand the effect that age, gender, family status, occupation, education, type of community where the person lives, political orientation, and religion have in the cross-European segments found and the proposed GMS strategy.

## Figures and Tables

**Table 1:** Sample size by country, Total population 15+.

COUNTRY	Number of Interviews	Population 15+
Austria	1006	7,554,711
Belgium	1041	9,693,779
Bulgaria	1026	6,537,535
Croatia	1010	3,796,476
Czech Republic	1068	9,238,431
Denmark	1017	4,838,729
Estonia	1005	1,160,064
Finland	1000	4,747,810
France	1013	54,097,255
Germany	1507	70,160,634
Greece	1014	9,937,810
Hungary	1030	8,781,161
Ireland	1078	3,592,162
Italy	1021	52,334,536
Latvia	1012	1,707,082
Lithuania	1004	2,513,384
Luxemburg	512	457,127
Malta	497	364,171
Netherlands	1017	13,979,215
Poland	1011	33,444,171
Portugal	1013	8,480,126
Republic of Cyprus	505	741,308
Romania	1025	16,852,701
Slovakia	1020	4,586,024
Slovenia	1016	1,760,032
Spain	1014	39,445,245
Sweden	1021	7,998,763
United Kingdom	1021	52,651,777
TOTAL	27,524	431,452,219

Source: Eurobarometer 91.2. European Commission [30,31] Table A1 (Appendix A) shows the variables selected from this Eurobarometer to perform the analysis and the sample statistical descriptives.

**Table 2:** Principal Components Analysis (Varimax Rotation).

Component # ^1^	Component Name ^1^	Original Variables with Significant Loadings	Percentage of Total Variance
C1	Vaccines not important	Vaccines important	9%
		Vaccines are rigorously tested before being authorized for use	
		Everybody needs to have routine vaccinations	
		Not getting vaccinated can lead to serious health issues	
		Vaccines are important to protect not only yourself but also others	
		Vaccination of other people is important to protect those that cannot be vaccinated	
C2	Infectious diseases kill	Flu is causing deaths in the EU nowadays	8%
		Measles is causing deaths in the EU nowadays	
		Polio is causing deaths in the EU nowadays	
		Hepatitis is causing deaths in the EU nowadays	
		Meningitis is causing deaths in the EU nowadays	
		Tetanus is causing deaths in the EU nowadays	
C3	Vaccines are dangerous	Vaccines overload and weaken the immune system	5%
		Vaccines can cause the disease against which they protect	
		Vaccines can often produce serious side-effects	
		Do not know at which level vaccination programs should be coordinated	
C4	Vaccine informed	Seen vaccine info in the last six months on TV	6%
		Seen vaccine info in the last six months on the radio	
		Seen vaccine info in the last six months in newspapers or magazines	
		Seen vaccine info in the last six months on online social networks	
		Seen vaccine info in the last six months on other Internet sites	
C5	Children vaccinated	Have a vaccination card for children	5%
		Children vaccinated in the last five years	
C6	Family & friends info	If you were looking for information about vaccination, you would consult family	3%
		If you were looking for information about vaccination, you would consult friends	
C7	European vaccination programs	Vaccination programs should be coordinated at European level	4%
		Vaccination programs should be coordinated at a national level	
		Vaccination programs should be coordinated at a regional or local level	
C8	Information insecurity	If you were looking for information about vaccination NONE of the following sources you would consult	4%
		If you were looking for information about vaccination DO NOT KNOW which of the following sources you would consult	
		Family is the source you trust the most for information on vaccination	
C9	Other sources of info	If you were looking for information about vaccination, you would consult other sources of information	3%
		In the past six months, you have seen, read or heard any information on vaccination in other media	
C10	Health system info	If you were looking for information about vaccination, you would consult other health care workers (nurses, specialist doctors, etc.)	3%
		If you were looking for information about vaccination, you would consult pharmacists	
		If you were looking for information about vaccination, you would consult the health authorities	
C11	Self-vaccinated	I got vaccinated in the last five years	4%
		I have a vaccination card	
		Tend to agree that not getting vaccinated can lead to serious health issues	
C12	Vaccination lack of knowledge	Do not know if you or someone in your family had any vaccinations in the last five years	4%
		Do not know if you have a vaccination card	
		Do not know if you were looking for information about vaccination, which of the following sources would you consult	
		Do not know if in the past six months you have seen, read or heard any information on vaccination in the media	
C13	Online media info	If you were looking for information about vaccination you would consult online social networks	4%
		If you were looking for information about vaccination you would consult other Internet sites	
		In the past six months you have seen, read or heard information on vaccination on online social networks	
		In the past six months you have seen, read or heard information on vaccination on another Internet sites	
C14	International vaccination programs	You think vaccination programs should be coordinated at an international level	3%
		You have a vaccination card	

^1^ The fourteen components obtained were labeled in two different ways. First, with a capital C followed by a number (Component #). This is the name given by R software when using the “psych” library for performing PCA analysis. We maintained these original names without any change for research reproducibility reasons. The other name of each the components were given in the need for obtaining a substantive interpretation of the pattern of the component loadings for the variables. Variables with higher significant factor loadings influenced the name selected to represent a factor to a greater extent [38].

**Table 3:** Bayesian Multilevel Multinomial Analysis. Unconditional Mean Model (log-odds).

	Posterior Mean ^1^	Posterior SD ^2^	Rhat ^3^
*Population-level effects (reference category: Pro-vaccinators)*			
Intercept Self-hesitants	−1.38	0.07	1.0
Intercept Social-hesitants	−1.85	0.11	1.0
Intercept Anti-vaccinators	−1.63	0.12	1.0
Intercept Alternative-hesitants	−3.20	0.11	1.0
Intercept Illiterate-hesitants	−2.90	0.17	1.0
Intercept Uninformed Anti-vaccinators	−3.54	0.21	1.0
*Country-level effects (reference category: Pro-vaccinators)*			
Intercept Self-hesitants	0.33	0.05	1.0
Intercept Social-hesitants	0.58	0.09	1.0
Intercept Anti-vaccinators	0.63	0.09	1.0
Intercept Alternative-hesitants	0.55	0.09	1.0
Intercept Illiterate-hesitants	0.89	0.14	1.0
Intercept Uninformed Anti-vaccinators	1.07	0.17	1.0

^1^ Mean of the posterior distribution using MCMC. ^2^ Standard deviation of the posterior distribution using MCMC. ^3^ Rhat is the potential scale reduction factor on split chains (at convergence, Rhat = 1).

**Table 4:** Cross-European Segments Towards Vaccination. Multinomial Logit Model (log-odds).

	*Dependent Variable (Reference Category Pro-Vaccinators):*					
	Self-Hesitants	Social Hesitants	Anti-Vaccinators	Alternative Hesitants	Illiterate Hesitants	Uninformed Anti-vaccinators
C1	−0.088	0.593 ***	2.475 ***	2.037 ***	1.899 ***	3.135 ***
	−0.058	−0.07	−0.055	−0.419	−0.215	−0.396
C2	−0.498 ***	0.05	−0.488 ***	−0.076	0.619 ***	0.454
	−0.041	−0.053	−0.044	−0.486	−0.231	−0.438
C3	0.308 ***	0.194 ***	1.141 ***	1.257 ***	1.591 ***	0.875 ***
	−0.049	−0.062	−0.046	−0.393	−0.194	−0.309
C4	−0.262 ***	−0.249 ***	−0.611 ***	−0.691	−2.597 ***	−1.617 ***
	−0.039	−0.056	−0.05	−0.555	−0.273	−0.554
C5	2.845 ***	0.157 ***	0.538 ***	−0.644	0.198	0.171
	−0.043	−0.051	−0.043	−0.663	−0.23	−0.464
C6	−0.569 ***	3.694 ***	−0.146 *	0.912 **	0.673 ***	−3.979 ***
	−0.067	−0.065	−0.077	−0.428	−0.223	−1.352
C7	−0.180 ***	−0.800 ***	−3.637 ***	−1.922 ***	−1.843 ***	−1.129 **
	−0.045	−0.062	−0.063	−0.498	−0.222	−0.441
C8	0.06	−0.720 ***	0.151 **	0.925	0.221	3.880 ***
	−0.057	−0.076	−0.068	−0.59	−0.321	−0.435
C9	−0.337 ***	0.537 ***	0.465 ***	5.333 ***	0.301	−0.702
	−0.106	−0.138	−0.123	−0.446	−0.515	−0.792
C10	−0.264 ***	−0.165 **	−0.729 ***	−1.181 *	−0.014	−3.343 ***
	−0.043	−0.068	−0.05	−0.608	−0.301	−0.761
C11	−0.378 ***	−0.101 **	−0.359 ***	−0.224	−0.851 ***	−1.261 **
	−0.033	−0.045	−0.039	−0.472	−0.225	−0.514
C12	0.214 **	0.033	0.607 ***	4.421 ***	6.999 ***	0.699
	−0.107	−0.141	−0.111	−0.382	−0.346	−0.48
C13	0.546 ***	−0.456 ***	−0.425 ***	−0.773	−0.704 ***	−3.534 ***
	−0.037	−0.056	−0.048	−0.487	−0.248	−1.045
C14	−0.366 ***	−0.468 ***	−1.092 ***	−1.366 ***	−2.323 ***	−1.013 **
	−0.036	−0.048	−0.041	−0.373	−0.202	−0.409
Constant	−3.303 ***	−3.934 ***	−3.039 ***	−9.268 ***	−8.875 ***	−12.241 ***
	−0.065	−0.083	−0.06	−0.687	−0.541	−1.153

Note: * *p* < 0.05, ** *p* < 0.01, *** *p* < 0.001.

## Data Availability

Publicly available datasets were analyzed in this study. This data can be found through GESIS (University of Cologne, Germany) at https://www.gesis.org/en/eurobarometer-data-service/search-data-access/data-access.

## Appendix A

**Table A1:** Sample descriptives.

Variable # ^1^	Variable	Response Categories	*n*	Sample Share
qc1	Diseases causing deaths in the EU nowadays	Flu	16,464	59.8
		Measles	10,547	38.3
		Polio	4808	17.5
		Hepatitis	11,323	41.1
		Meningitis	14,190	51.6
		Tetanus	6315	22.9
		None of them	2348	8.5
		Don’t Know (DK)	1728	6.3
qc2	Vaccines effective	Yes, definitely	13,972	50.8
		Yes, probably	9450	34.3
		No, probably not	1664	6
		No, not at all	870	3.2
		It depends on the disease	1011	3.7
		Don’t Know (DK)	557	2
qc3	Vaccinations in family last five years	Yes, yourself	11,820	42.9
		Yes, your children	7164	26
		Yes, someone else	5521	19.1
		No	9683	35.2
		Don’t Know (DK)	315	1.1
qc6	Have vaccination card	Yes, for yourself	11,754	42.7
		Yes, for your children	6813	24.8
		No	11,978	43.5
		Don’t Know (DK)	651	2.4
qc7_1	Vaccines affect the immune system	TRUE	8849	32.2
		FALSE	14,461	52.5
		Do not Know (DK)	4214	15.3
qc7_2	Vaccines cause diseases	TRUE	10,669	38.8
		FALSE	13,066	47.5
		Don’t Know (DK)	3789	13.8
qc7_3	Vaccines side-effects	TRUE	13,617	49.5
		FALSE	10,735	39
		Don’t Know (DK)	3172	11.5
qc7_4	Vaccines tested	TRUE	21,884	79.5
		FALSE	2736	9.9
		Don’t Know (DK)	2904	10.6
qc8_1	Routine vaccination important	Totally agree	14,222	51.7
		Tend to agree	8776	31.9
		Tend to disagree	2670	9.7
		Totally disagree	890	3.2
		Don’t Know (DK)	966	3.5
qc8_2	Vaccines only for children	Totally agree	3643	13.2
		Tend to agree	4551	16.5
		Tend to disagree	8351	30.3
		Totally disagree	10,270	37.3
		Don’t Know (DK)	709	2.6
qc8_3	Not vaccinated serious health issues	Totally agree	13,065	47.5
		Tend to agree	9371	34
		Tend to disagree	3008	10.9
		Totally disagree	932	3.4
		Don’t Know (DK)	1148	4.2
qc8_4	Vaccines’ importance for self and others	Totally agree	15,284	55.5
		Tend to agree	8956	32.5
		Tend to disagree	1819	6.6
		Totally disagree	577	2.1
		Don’t Know (DK)	888	3.2
qc8_5	Vaccines’ importance for non-vaccinated	Totally agree	14,505	52.7
		Tend to agree	9296	33.8
		Tend to disagree	1786	6.5
		Totally disagree	598	2.2
		Don’t Know (DK)	1339	4.9
qc9	Vaccine info sources	Family	2627	9.5
		Friends	1351	4.9
		Your general practitioner, a doctor, or a pediatrician	21,765	79.1
		Other health care workers (nurses, specialist doctors, etc.)	8647	31.4
		Pharmacists	5103	18.5
		Online social networks	1632	5.9
		Other Internet sites	3539	12.9
		The health authorities	7570	27.5
		Other (SPONTANEOUS)	298	1.1
		None (SPONTANEOUS)	504	1.8
		Don’t Know (DK)	238	0.9
qc10	Most trusted vaccine info source	Family	845	3.1
		Friends	355	1.3
		Your general practitioner, a doctor, or a pediatrician	17,521	63.7
		Other health care workers (nurses, specialist doctors, etc.)	2695	9.8
		Pharmacists	928	3.4
		Online social networks	285	1
		Other Internet sites	553	2
		The health authorities	3323	12.1
		Other (SPONTANEOUS)	74	0.3
		None (SPONTANEOUS)	576	2.1
		Don’t Know (DK)	369	1.3
qc11	Vaccine programs’ coordination level	At international level	8911	32.4
		At European level	8399	30.5
		At a national level	4526	42.4
		At regional or local level	2512	9.1
		There should be no vaccination programs, it is a personal choice		
		Don’t Know (DK)	1305	4.7
qc12	Media info on vaccine last six months	No	8052	29.3
		Yes, on TV	15,447	56.1
		Yes, on the radio	4775	17.3
		Yes, in newspapers or magazines	5771	21
		Yes, on online social networks	3571	13
		Yes, on other Internet sites	2835	10.3
		Other (SPONTANEOUS)	433	1.6
		Don’t Know (DK)	490	1.8

^1^ We kept the question number used in the Eurobarometer in order to be easily identifiable in the original questionnaire codebook. Source: Eurobarometer 91.2. European Commision [30].

## Appendix B

**Table A2:** PCA components loadings (VARIMAX rotation).

Components	Components (Eigen Values)													
	C1 (5.00)	C2 (4.43)	C4 (3.29)	C5 (2.53)	C3 (2.47)	C12 (2.30)	C8 (2.24)	C11 (2.16)	C7 (2.14)	C13 (1.86)	C6 (1.71)	C10 (1.69)	C9 (1.62)	C14 (1.33)
Question	Vaccines are Not Important	Infectious Diseases Kill	Vaccine Informed	Children Vaccinated	Vaccines Are Dangerous	Vaccination Lack of Knowledge	Information Insecurity	Self Vaccinated	European Vaccination Programs	Online Media Info	Family & Friends Info	Health System Info	Other Sources of Information	International Vaccination Programs
qc1.1		0.7												
qc1.2		0.7												
qc1.3		0.8												
qc1.4		0.7												
qc1.5		0.7												
qc1.6		0.8												
qc1.7														
qc1.8														
qc2	0.6													
qc3.1								0.7						
qc3.2				0.8										
qc3.3								0.7						
qc3.4														
qc3.5						0.7								
qc6.1								0.5						0,5
qc6.2				0.9										
qc6.3														
qc6.4						0.7								
qc7_1					0.8									
qc7_2					0.8									
qc7_3					0.8									
qc7_4	0.5													
qc8_1	0.8													
qc8_2								0.5						
qc8_3	0.8													
qc8_4	0.9													
qc8_5	0.8													
qc9.1											0.8			
qc9.2											0.8			
qc9.3														
qc9.4												0.7		
qc9.5												0.5		
qc9.6										0.6				
qc9.7										0.8				
qc9.8												0.8		
qc9.9													0.7	
qc9.10	0.5						0.5							
qc9.11	0.4					0.4	0.5							
qc.10							0.9							
qc11.1														0,6
qc11.2									0.6					
qc11.3									0.8					
qc11.4									0.8					
qc11.5	0.6													
qc11.6					0.4									
qc12.1														
qc12.2			0.8											
qc12.3			0.9											
qc12.4			0.7											
qc12.5			0.6							0.5				
qc12.6			0.5							0.5				
qc12.7													0.8	
qc12.8						0.5								

## Appendix C

**Table A3:** ANOVA (Tukey HSD). Segments’ mean differences.

Title Vaccine-Hesitancy Segments	Vaccines Are Not Important	Infectious Diseases Kill	Vaccine Informed	Children Vaccinated	Vaccines Are Dangerous	Vaccination Lack of Knowledge	Information Insecurity
Self-hesitants/Pro-vaccinators	0.05 **	−0.08 ***	−0.03	1.82 ***	0.10 ***	0	−0.01
Social hesitants/Pro-vaccinators	0.27 ***	0.02	0.04	0.28 ***	0.04	−0.12 ***	0.22 ***
Anti-vaccinators/Pro-vaccinators	1.08 ***	−0.08 ***	−0.04	0.32 ***	0.35 ***	−0.29 ***	−0.40 ***
Alternative hesitants/Pro-vaccinators	0.35 ***	−0.05	−0.27 ***	0.37 ***	0.05	−0.08 **	0.14 ***
Illiterate hesitants/Pro-vaccinators	0.40 ***	−0.05	−0.4 ***	0.24 ***	0.20 ***	3.00 ***	0.05
Uninformed anti-vaccinators/Pro-vaccinators	1.58 ***	−0.13 **	0.02	0.13 ***	0.14 **	−0.67 ***	2.84 ***
Social hesitants/Self-hesitants	0.21 ***	0.09 ***	0.06*	−1.55 ***	−0.06	−0.12 ***	0.23 ***
Anti-vaccinators/Self-hesitants	1.03 ***	0.00	−0.01	−1.5 ***	0.25 ***	−0.29 ***	−0.39 ***
Alternative hesitants/Self-hesitants	0.30 ***	0.03	−0.24 ***	−1.45 ***	−0.05	−0.08 *	0.15 ***
Illiterate hesitants/Self-hesitants	0.35 ***	0.03	−0.37 ***	−1.59 ***	0.10 *	3.00 ***	0.06
Uninformed anti-vaccinators/Self-hesitants	1.53 ***	−0.05	0.05	−1.69 ***	0.04	−0.67 ***	2.85 ***
Anti-vaccinators/Social hesitants	0.82 ***	−0.09 ***	−0.08 **	0.04	0.31 ***	−0.16 ***	−0.62 ***
Alternative hesitants/Social hesitants	0.08	−0.06	−0.31 ***	0.09	0.01	0.04	−0.09
Illiterate hesitants-Social hesitants	0.14 ***	−0.07	−0.44 ***	−0.04	0.16	3.13 ***	−0.17 ***
Uninformed anti-vaccinators/Social hesitants	1.32 ***	−0.14 ***	−0.02	−0.15 ***	0.1	−0.55 ***	2.61 ***
Alternative hesitants/Anti-vaccinators	−0.073 ***	0.03	−0.23 ***	0.05	−0.30 ***	0.20 ***	0.53 ***
Illiterate hesitants-Anti-vaccinators	−0.68 ***	0.02	−0.36 ***	−0.08 *	−0.15 ***	3.29 ***	0.45 ***
Uninformed anti-vaccinators/Anti-vaccinators	0.50 ***	−0.05	0.06	−0.19 *	−0.21 ***	−0.38 ***	3.23 ***
Illiterate hesitants/Alternative hesitants	0.05	0.00	−0.13 *	−0.13 ***	0.15 *	3.08 ***	−0.09
Uninformed anti-vaccinators/Alternative hesitants	1.23 ***	−0.08	0.29 ***	−0.24 **	0.09	−0.59 ***	2.70 ***
Uninformed anti-vaccinators/Illiterate hesitants	1.18 ***	−0.08	0.42 ***	−0.11 ***	−0.06	−3.67 ***	2.78 ***
Self-hesitants/Pro-vaccinators	−0.34 ***	0.01	0.24 ***	−0.07 ***	−0.13 ***	−0.04 ***	−0.14 ***
Social hesitants/Pro-vaccinators	0	−0.13 ***	−0.23 ***	2.15 ***	−0.30 ***	−0.04 ***	−0.05
Anti-vaccinators/Pro-vaccinators	−0.14 ***	−1.09 ***	0.06 *	0.05 ***	0	−0.07 ***	−0.21 ***
Alternative hesitants/Pro-vaccinators	−0.04	−0.07	−0.22 ***	0.34 ***	−0.13 **	4.90 ***	−0.11
Illiterate hesitants/Pro-vaccinators	−0.20 ***	−0.27 ***	0.13 ***	0.28 ***	0.15 ***	−0.06 ***	−0.69 ***
Uninformed anti-vaccinators/Pro-vaccinators	−0.01	−0.22 ***	−0.81 ***	−0.59 ***	−1.47 ***	−0.72 ***	−0.01
Social hesitants/Self-hesitants	0.34 ***	−0.14 ***	−0.47 ***	2.23 ***	−0.16 ***	0	0.10 **
Anti-vaccinators/Self-hesitants	0.20 ***	−1.10 ***	−0.18 ***	0.13 ***	0.13 ***	−0.03 **	−0.07 *
Alternative hesitants/Self-hesitants	0.30 ***	−0.08	−0.46 ***	0.42 ***	0	4.95 ***	0.03
Illiterate hesitants/Self-hesitants	0.14 **	−0.27 ***	−0.11 **	0.35 ***	0.28 ***	−0.02	−0.54 ***
Uninformed anti-vaccinators/Self-hesitants	0.32 ***	−0.22 ***	−1.05 ***	−0.51 ***	−1.34 ***	−0.67 ***	0.13 *
Anti-vaccinators/Social hesitants	−0.14 ***	−0.96 ***	0.29 ***	−2.10 ***	0.30 ***	−0.04	−0.17 ***
Alternative hesitants/Social hesitants	−0.04	0.06	0.01	−1.81 ***	0.16 ***	4.94 ***	−0.06
Illiterate hesitants-Social hesitants	−0.20 ***	−0.14 ***	0.35 ***	−1.87 ***	0.45 ***	−0.03 **	−0.64 ***
Uninformed anti-vaccinators/Social hesitants	−0.01	−0.09	−0.59 ***	−2.74 ***	−1.18 ***	−0.68 ***	0.04
Alternative hesitants/Anti-vaccinators	0.11	1.02 ***	−0.28 ***	0.29 ***	−0.13 **	4.98 ***	0.10
Illiterate hesitants-Anti-vaccinators	−0.06	0.83 ***	0.07	0.23 ***	0.15 ***	0.01	−0.47 ***
Uninformed anti-vaccinators/Anti-vaccinators	0.13 *	0.88 ***	−0.87 ***	−0.64 ***	−1.47 ***	−0.64 ***	0.20 ***
Illiterate hesitants/Alternative hesitants	−0.17 **	−0.19 ***	0.35 ***	−0.06	0.28 ***	−4.97 ***	−0.58 ***
Uninformed anti-vaccinators/Alternative hesitants	0.02	−0.14 *	−0.59 ***	−0.93 ***	−1.34 ***	−5.62 ***	0.10
Uninformed anti-vaccinators/Illiterate hesitants	0.19 **	0.05	−0.94 ***	−0.87 ***	−1.63 ***	−0.65 ***	0.68 ***

Note: * *p* < 0.05, ** *p* < 0.01, *** *p* < 0.001.
